# Supplementary material for: ZBTB48 is both a vertebrate telomere‐binding protein and a transcriptional activator
Source: EMBO Rep. 2017 May 12;18(6):929–46. doi: 10.15252/embr.201744095 (PMC5452029; doi:10.15252/embr.201744095)
Supplement: Supplementary file 1 — Expanded View Figure PDF [file EMBR-18-929-s001.pdf]

Expanded View Figures

Figure EV1. ZBTB48 ZnF11 is necessary to bind to telomeres.

A Sequence-specific DNA pull-downs with either telomeric (TTAGGG) or a control sequence (GTGAGT) for FLAG-ZBTB48 WT, domain deletion constructs for different zinc finger and combinations of deletion constructs with ZnF10 or 11 point mutants. Domain structures are indicated on the right.

B Sequence-specific DNA pull-downs for FLAG-ZBTB48 WT and ZnF11 point mutant for telomeric repeat sequences of different phyla (green) and their respective scrambled controls (blue).

C Protein expression analysis of ZBTB48 by Western blot for the cell lines used in this study. GAPDH serves as a loading control.

D IF stainings for exogenous FLAG-ZBTB48 WT and point mutants for ZnF10 and ZnF11 in U2OS cells. The same analysis as in Fig 1E was performed and average co-localization frequencies are shown (n = 24–37 cells).

E Co-localization analysis of endogenous ZBTB48 or exogenous FLAG-ZBTB48 WT with TRF2 in HeLa cells by immunofluorescence (IF) staining. A representative image illustrating the co-localization between ZBTB48 or FLAG-ZBTB48 WT (green) and TRF2 (red) as a marker for telomeres is shown with DAPI (blue) used as a nuclear counterstain. Co-localization events are indicated by white arrows. The quantification of frequency of co-localization events (right) was done after 3D reconstruction of the acquired z-stacks (n = 30 cells).

F Co-localization analysis of endogenous ZBTB48 or exogenous FLAG-ZBTB48 WT with TRF2 in HeLa 1.3 cells by immunofluorescence (IF) staining analogous to (E) (n = 30 cells).

G Co-localization analysis of endogenous ZBTB48 or exogenous FLAG-ZBTB48 WT with TRF2 in HT1080 super-telomerase cells by immunofluorescence (IF) staining analogous to (E) (n = 30 cells).

Data information: (D–G) Scale bars represent 5 μm. Error bars indicate standard deviations, and P-values are based on Student's t-test.
Source data are available online for this figure.

© 2017 The Authors

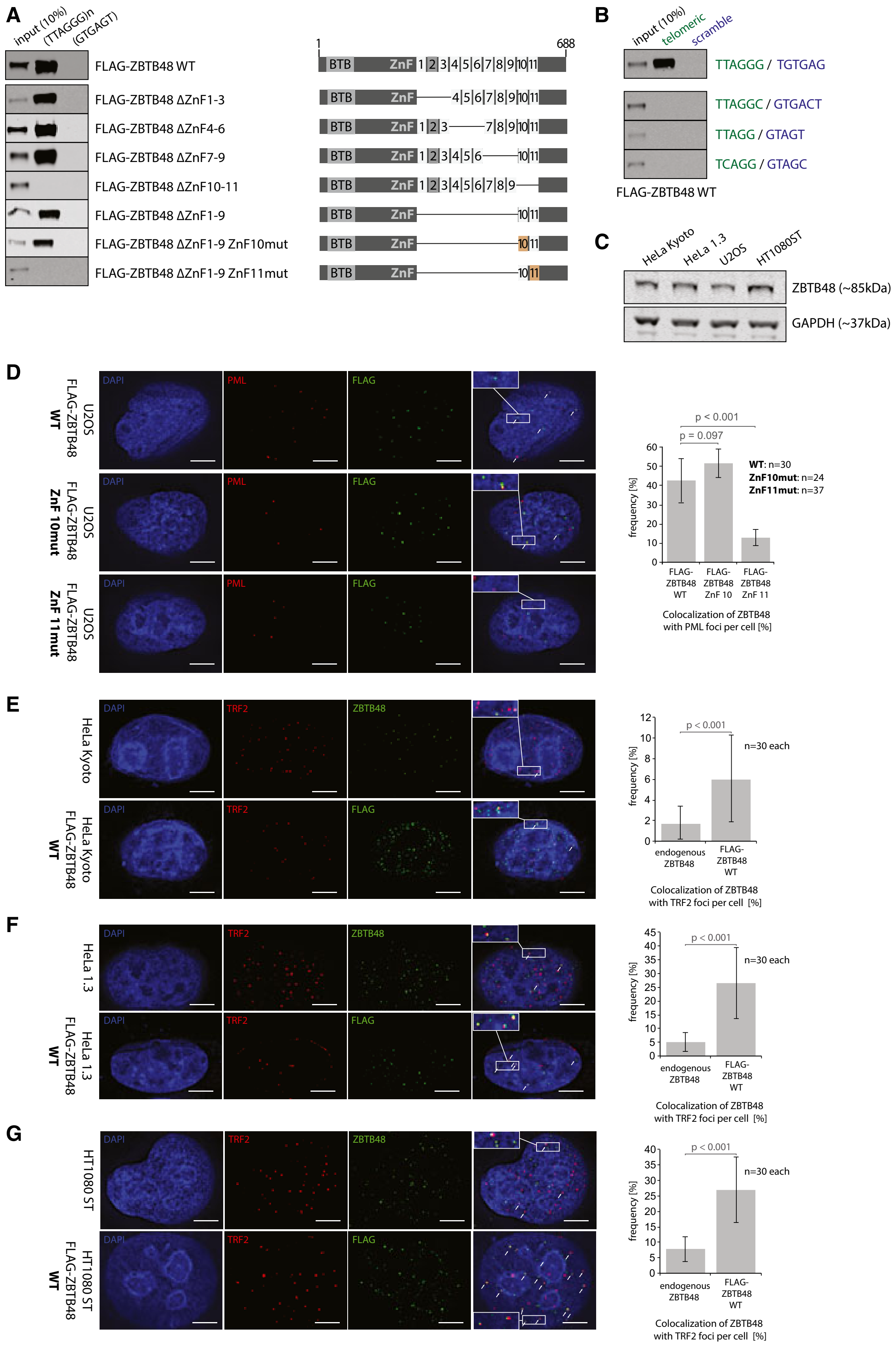

Figure EV1.

 © 2017 The Authors

Figure EV2. ZBTB48 and HOT1 KO clones show depleted expression.

A Depiction of the ZBTB48 TALEN binding sites located in exon 2.
B Genotypes of HeLa and U2OS WT and ZBTB48 KO clones. The grey boxes represent the TALEN binding sites; insertions (blue) and deletions (red) are marked. The numbers in brackets represent the numbers of clones found with the specific genotype.
C Gel image of T7E1 assay showing ZBTB48 TALEN activity in HeLa cells.
D Western blot confirmation of depleted ZBTB48 expression in each five HeLa and U2OS ZBTB48 KO clones compared to parental cells.
E IF confirmation of depleted ZBTB48 expression (green) with DAPI (blue) as nuclear counterstain in U2OS ZBTB48 KO cells. ZBTB48 signals are reduced to background levels. Scale bars represent 5 μm.
F Depiction of the HOT1 TALEN binding sites located in exon 3.
G Genotypes of HeLa and U2OS WT and HOT1 KO clones. The grey boxes represent the TALEN binding sites; insertions (blue) and deletions (red) are marked. The numbers in brackets represent the numbers of clones found with the specific genotype.
H Gel image of T7E1 assay showing HOT1 TALEN activity in HeLa cells.
I IF confirmation of depleted HOT1 expression (green) with DAPI (blue) as nuclear counterstain in a representative HeLa HOT1 KO clone. Scale bars represent 5 μm.

Source data are available online for this figure.

© 2017 The Authors

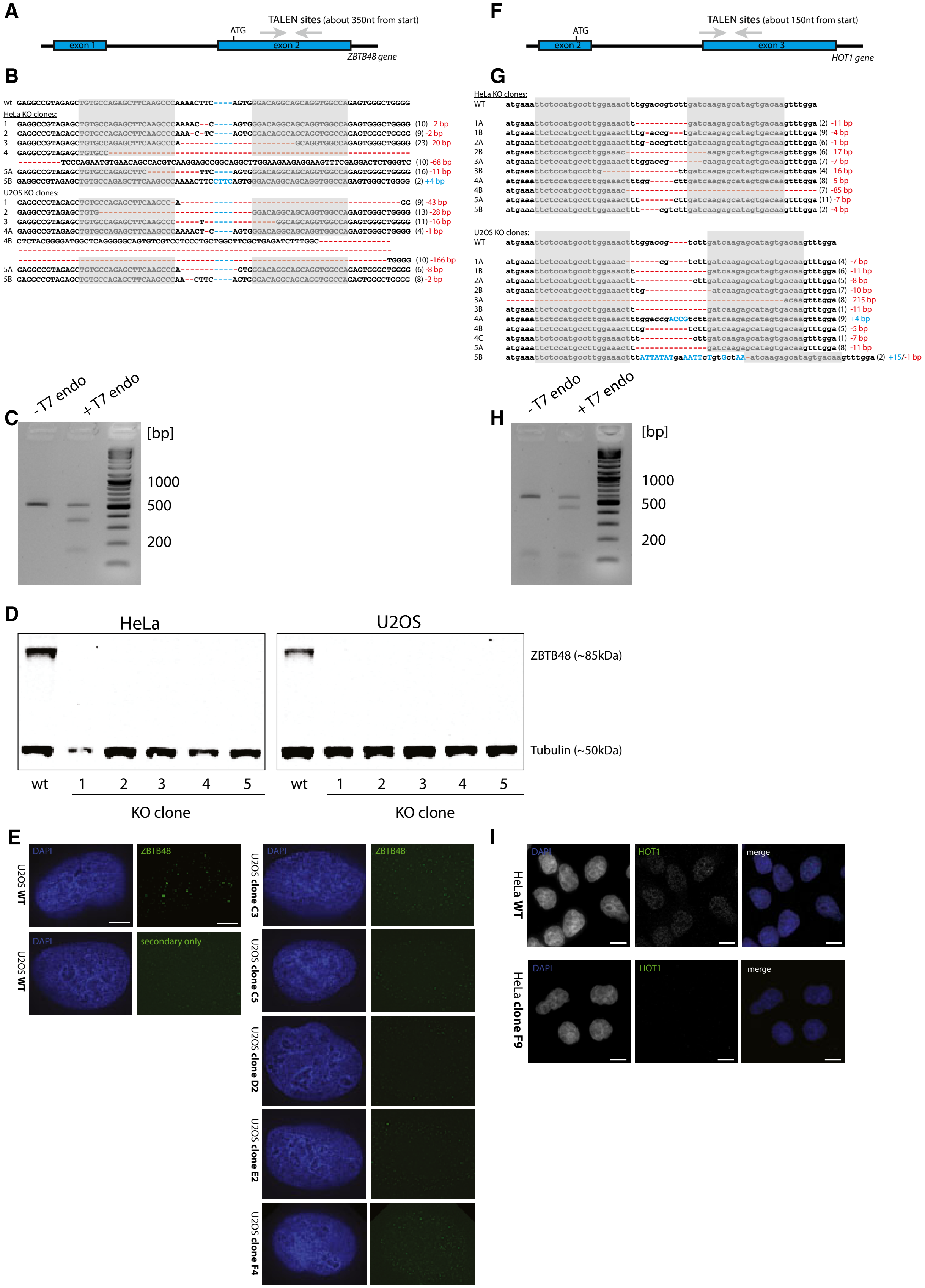

Figure EV2.

 © 2017 The Authors

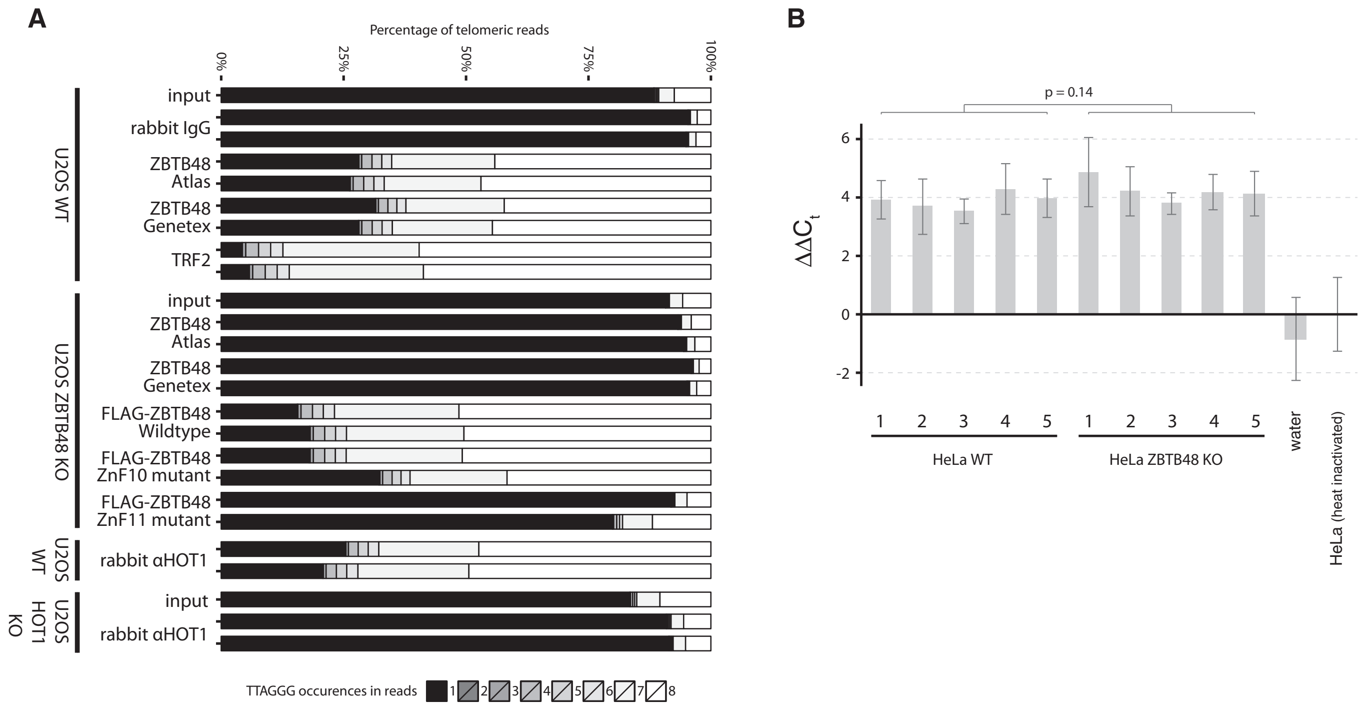

Figure EV3. ZBTB48 is a direct telomere-binding protein but TRAP activity is not affected in ZBTB48 KO clones.

A TTAGGG content of telomeric reads in ChIPseq samples from U2OS WT, U2OS ZBTB48 KO and U2OS HOT1 KO clones using IgG, TRF2, ZBTB48 Atlas, ZBTB48 GeneTex and HOT1 antibodies as well as FLAG antibody for FLAG-ZBTB48 WT and point mutants for ZnF10 and ZnF11. The percentage contribution to all reads containing 1–8× TTAGGG repeats is shown. All reactions were performed in technical replicates on two independent WT and KO clones each. These reactions are biological replicates of Fig 2A, performed with independent U2OS ZBTB48 WT and KO clones for ZBTB48 and with a second HOT1 antibody for HOT1.

B Telomerase activity was determined based on a quantitative TRAP assay. Heat-inactivated HeLa extracts were used as a threshold to determine non-specific background signal. Differences in C_t values ($\Delta\Delta C_t$) from the quantitative PCR measurements are displayed relative to the heat-inactivation control. Error bars represent standard deviations (n = 3). The P-value is based on Student's t-test.

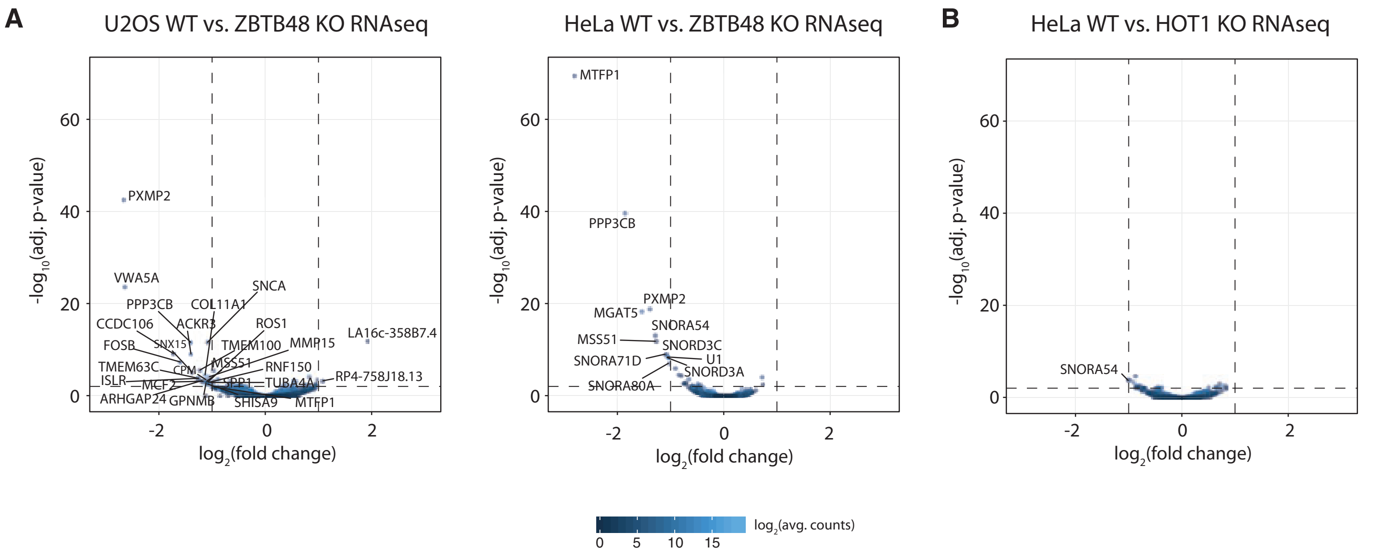

Figure EV4. ZBTB48 acts as a transcription factor in contrast to HOT1.

A Differential expression analysis of the RNA sequencing (RNAseq) gene quantitation, comparing each five WT and ZBTB48 KO clones for U2OS (left) and HeLa (right). Cut-offs for significant differential expression were set to $\log_2$(fold change) > |1| and $-\log_{10}$(adjusted *P*-value) > 2 (FDR < 0.01). These data are analogous to Fig 5A, but without cropping the *y*-axis.

B Differential expression analysis of the RNA sequencing (RNAseq) data comparing each five HeLa WT and HOT1 KO clones. Cut-offs for significant differential expression were set to $\log_2$(fold change) > |1| and $-\log_{10}$(adjusted *P*-value) > 2 (FDR < 0.01).

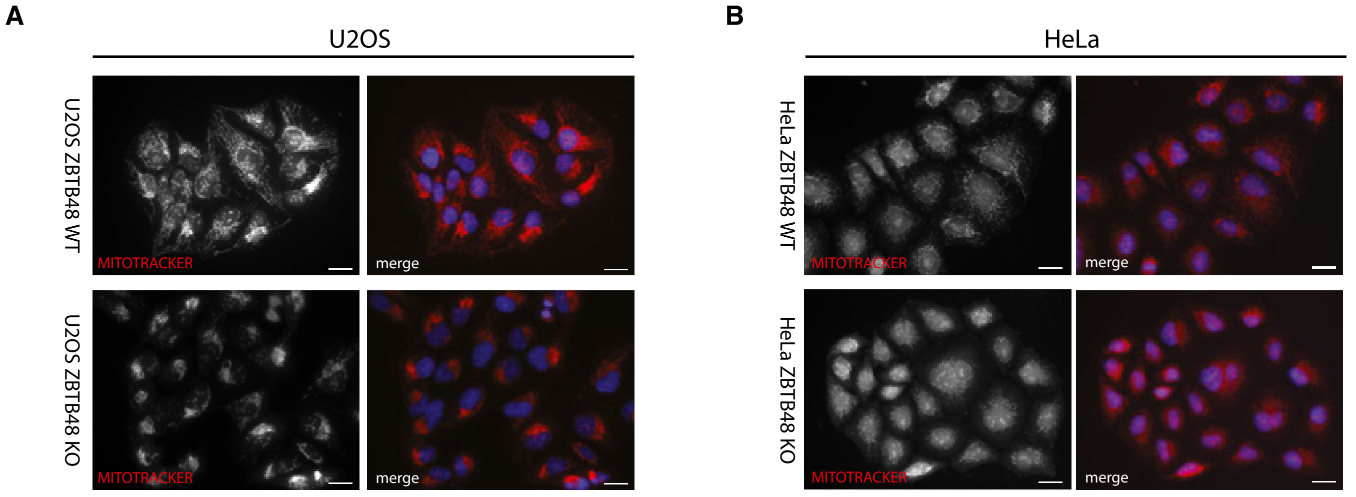

Figure EV5. ZBTB48-dependent loss of MTFP1 phenocopies MTFP1 depletion.

A Fluorescence microscopy analysis of the structure and localization of the mitochondrial network in HeLa WT and ZBTB48 KO clones. Mitochondria are marked with the MitoTracker dye (red), and nuclei are counterstained with DAPI (blue). Scale bars represent 20 µm.

B The same analysis as in (A) for U2OS WT and ZBTB48 KO clones.

 © 2017 The Authors
