# Supplementary material for: ZBTB48 is both a vertebrate telomere‐binding protein and a transcriptional activator
Source: EMBO Rep. 2017 May 12;18(6):929–46. doi: 10.15252/embr.201744095 (PMC5452029; doi:10.15252/embr.201744095)
Supplement: Supplementary file 8 — Movie EV1 [file EMBR-18-929-s008.zip › Movie_EV1_legend.docx]

**Movie EV1: ZBTB48-dependent loss of MTFP1 pheno-copies MTFP1 depletion**

Super-resolution fluorescence microscopy analysis of the structure and localization of the mitochondrial network in HeLa ZBTB48 WT. Mitochondria are marked with the mitotracker dye (red) and nuclei are counterstained with DAPI (blue). This is the 3D reconstructed image for which the Z projection is shown in **Fig 6B**.
