# Supplementary figures and images for: ZBTB48 is both a vertebrate telomere‐binding protein and a transcriptional activator
Source: EMBO Rep. 2017 May 12;18(6):929–46. doi: 10.15252/embr.201744095 (PMC5452029; doi:10.15252/embr.201744095)

Source Data to Fig EV1

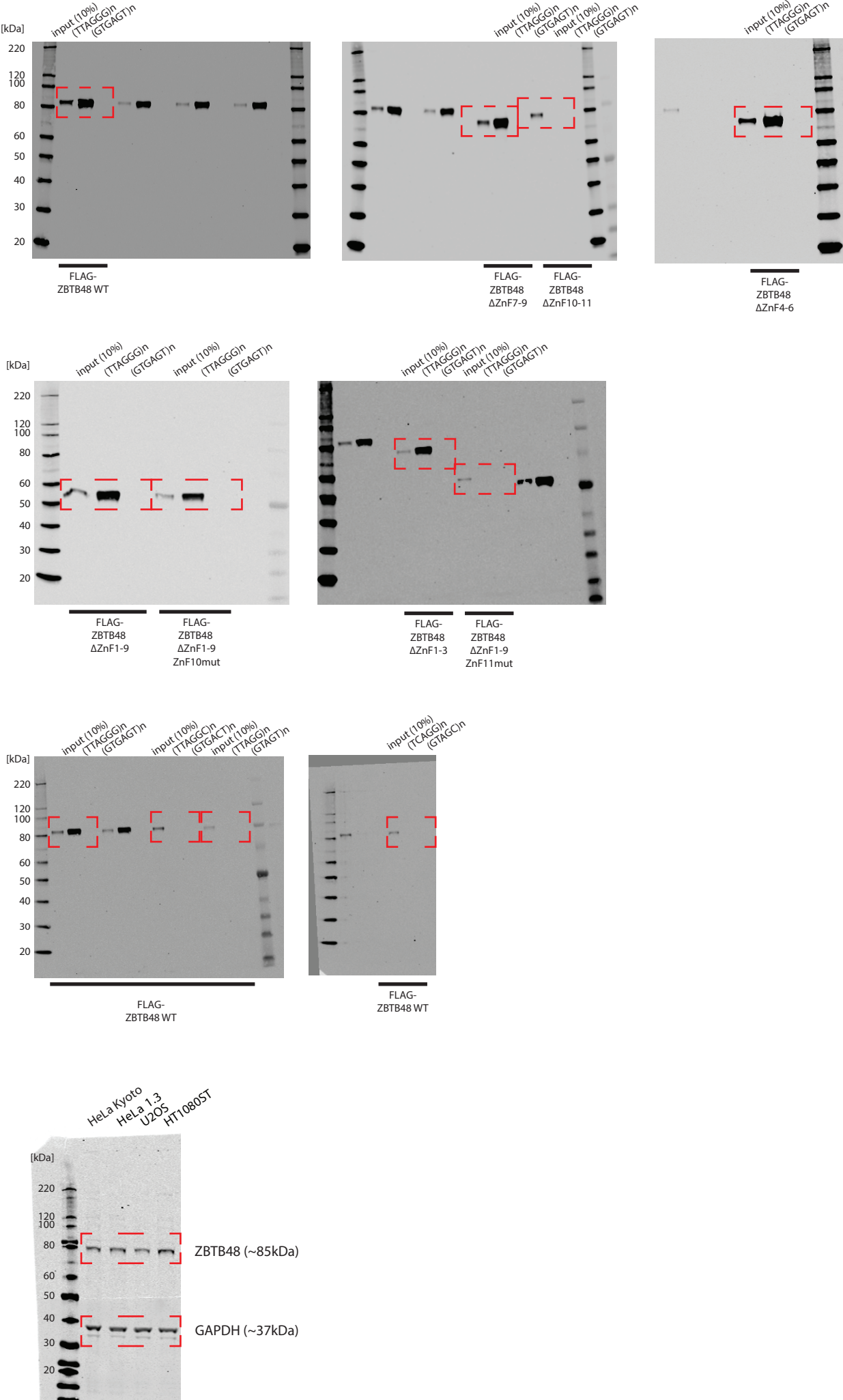

Supplement: Supplementary file 10 — Source Data for Expanded View [file EMBR-18-929-s011.zip › Source_Data_fig_EV1.pdf]

Source Data to Fig EV2

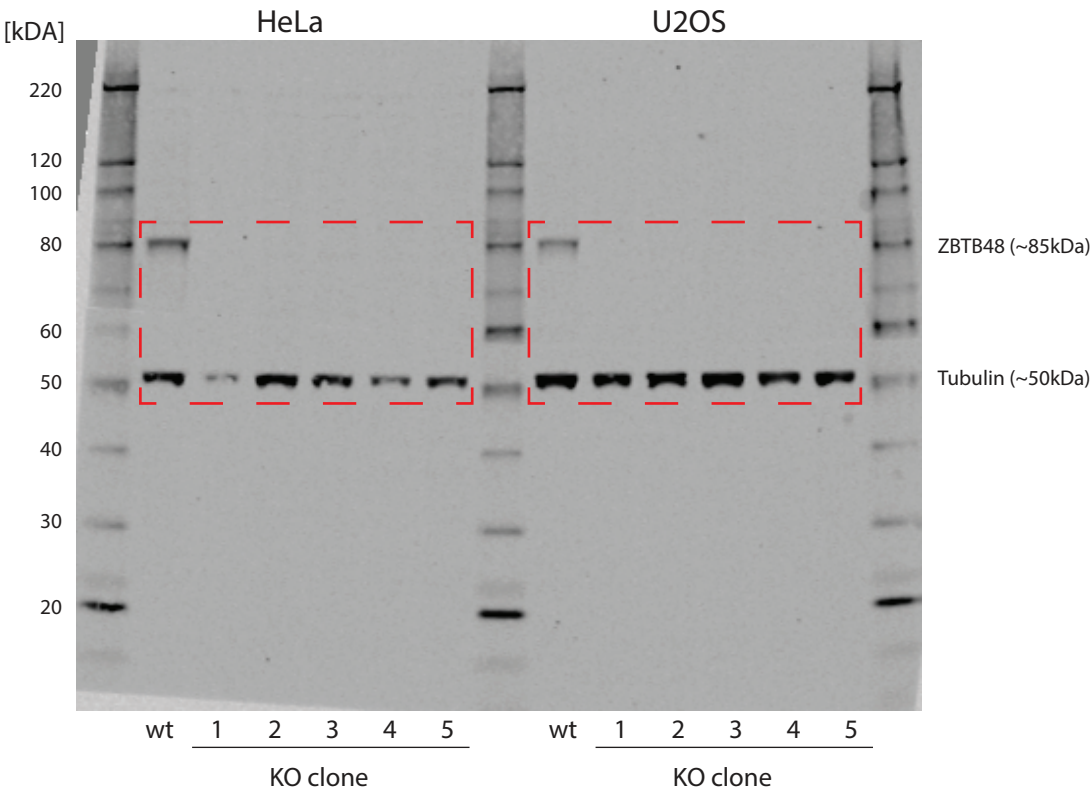

Supplement: Supplementary file 10 — Source Data for Expanded View [file EMBR-18-929-s011.zip › Source_Data_fig_EV2.pdf]
